# Multiple datasets to explore the molecular mechanism of sepsis

**DOI:** 10.1186/s12863-022-01078-2

**Published:** 2022-08-15

**Authors:** Shuang Lin, Bin Luo, Junqi Ma

**Affiliations:** 1 Emergency Department, Fourth Affiliated Hospital of Xinjiang Medical University, Shayibake District, No. 116, Huanghe Road, Urumqi, 830000 Xinjiang Uygur Autonomous Region China; 2 Department of Critical Care Medicine, Fifth Affiliated Hospital of Xinjiang Medical University, Urumqi, 830000 Xinjiang Uygur Autonomous Region China

**Keywords:** Sepsis, Immunity, Genes

## Abstract

**Background:**

This study aimed to identify potential biomarkers, by means of bioinformatics, affecting the occurrence and development of septic shock.

**Methods:**

Download GSE131761 septic shock data set from NCBI geo database, including 33 control samples and 81 septic shock samples. GSE131761 and sequencing data were used to identify and analyze differentially expressed genes in septic shock patients and normal subjects. In addition, with sequencing data as training set and GSE131761 as validation set, a diagnostic model was established by lasso regression to identify key genes. ROC curve verified the stability of the model. Finally, immune infiltration analysis, enrichment analysis, transcriptional regulation analysis and correlation analysis of key genes were carried out to understand the potential molecular mechanism of key genes affecting septic shock.

**Results:**

A total of 292 differential genes were screened out from the self-test data, 294 differential genes were screened out by GSE131761, Lasso regression was performed on the intersection genes of the two, a diagnostic model was constructed, and 5 genes were identified as biomarkers of septic shock. These 5 genes were SIGLEC10, VSTM1, GYPB, OPTN, and GIMAP7. The five key genes were strongly correlated with immune cells, and the ROC results showed that the five genes had good predictive performance on the occurrence and development of diseases. In addition, the key genes were strongly correlated with immune regulatory genes.

**Conclusion:**

In this study, a series of algorithms were used to identify five key genes that are associated with septic shock, which may become potential candidate targets for septic shock diagnosis and treatment.

**Trial registration:**

Approval number:2019XE0149-1.

## Background

Sepsis (sepsis) refers to life-threatening organ dysfunction caused by an imbalance in the host response caused by infection, and septic shock is a kind of sepsis [1]. The excessively activated inflammatory response in the early stage of sepsis causes serious damage to the body and even leads to organ failure and septic shock [2]. In recent years, there have been many basic and clinical studies on sepsis at home and abroad, but few studies have fully elucidated the specific pathogenesis of septic shock. In previous genomic and transcriptomic studies, many studies have focused on the differences between septic patients and healthy individuals [3], but there are insufficient studies on the mechanism of action of septic shock. Studies have shown that early warning and identification of risk factors for patients with sepsis can lead to faster and more accurate standardized treatment, which is helpful for the diagnosis, treatment and prognosis of sepsis [4].

Immune disorder is an important mechanism of sepsis. Sepsis is the result of the interaction between the body and pathogens. The body’s immune response to infection occurs through two pathways, the innate immune system and the adaptive immune system. When sepsis occurs, pathogens invade the body, the innate immune system responds to microbial components, a variety of inflammatory cells are activated, and a large number of proinflammatory factors and inflammatory mediators are released. These inflammatory factors produce a cascade effect through their own positive feedback, resulting in an excessive inflammatory response. At the same time, the release of anti-inflammatory factors is also increased in a compensatory manner, proinflammatory/anti-inflammatory responses coexist and oppose each other, and the body experiences a complex immune dynamic cell apoptosis imbalance and enters an immunosuppressive state [5]. The innate immune system recognizes pathogenic microorganisms through Toll-like receptors (TLRs), and the signalling pathways mediated by them play an important role in the development of sepsis and septic shock. The mechanisms of immunosuppression in sepsis include immune cell exhaustion and apoptosis, including CD4 + T cells, CD8 + T cells, NK cells, neutrophils, dendritic cells, macrophages, and monocytes, among which T cells are the most affected. The effector function of T cells is impaired, the antigen presentation ability is impaired, and the secretion of cytokines is dysregulated [5–7].

This study focused on elucidating the molecular mechanism of the development of septic shock in patients with sepsis. Differentially expressed genes were screened, and the Gene Ontology (GO) and Kyoto Encyclopedia of Genes and Genomes (KEGG) databases were used for enrichment analysis. Analysis, detection of signalling pathways related to the occurrence and development of sepsis, and analysis of gene expression differences were performed to provide a mechanistic understanding of the signalling pathways involved in identifying and responding to septic shock in sepsis patients. Further prevention and treatment of septic shock can provide early diagnosis and treatment strategies.

## Materials and methods

### Gene chip data download and ethics

A total of 19 patients were included in the self-assessment data, including 10 patients with no septic shock and 9 patients with septic shock. The mRNA transcriptome of peripheral whole blood samples was sequenced, and the data were analyzed to find out the differential genes. The differentially expressed genes were enriched and analyzed in the gene ontology (go) and the Kyoto Encyclopedia of genes and genomes (KEGG) databases. After protein network interaction analysis, the key genes were screened, and the samples were further expanded to twice the number of sequenced cases for qPCR verification.

The Series Matrix File data file of GSE131761 was downloaded from the NCBI GEO public database, and the analysis file was GPL13497. A total of 114 groups of patients were included in the expression profile data, including 33 patients with no septic shock and 81 patients with septic shock.

### Functional annotation of GO and KEGG

Differentially expressed genes were functionally annotated using the R package “ClusterProfiler” to comprehensively explore the functional relevance of these genes. Gene Ontology (GO) and Kyoto Encyclopedia of Genes and Genomes (KEGG) were used to assess related functional categories. GO and KEGG enriched pathways with both *p* values and q-values less than 0.05 were considered significant categories.

### WGCNA [8]

By constructing a weighted gene coexpression network, we searched for coexpressed gene modules and explored the relationship between gene networks and phenotypes, as well as the core genes in the network. The coexpression network of all genes in the GSE131761 dataset was constructed using the WGCNA-R package, and the genes with the top 5000 variance were screened with this algorithm for further analysis, where the soft threshold was set to 4. The weighted adjacency matrix was transformed into a topological overlap matrix (TOM) to estimate the degree of network connectivity, and the hierarchical clustering method was used to construct the clustering tree structure of the TOM matrix. Different branches of the clustering tree represented different gene modules, and different colours represented different modules. Based on the weighted correlation coefficient of genes, the genes were classified according to their expression patterns, genes with similar patterns were grouped into one module, and tens of thousands of genes were divided into multiple modules by gene expression patterns.

### Model construction

Differentially expressed genes were selected, and lasso regression was used to further construct a prognostic correlation model. After incorporating the expression values for each specific gene, a scoring formula for each patient was constructed and weighted by its estimated regression coefficients in a lasso regression analysis. According to the scoring formula, ROC curves were used to study the accuracy of model prediction.

### Analysis of immune cell infiltration

CIBERSORT is a widely used tool for quantifying immune cell content [9]. The method is based on the principle of support vector regression to perform deconvolution analysis on the expression matrix of immune cell subtypes. It contains 547 biomarkers that distinguish 22 human immune cell phenotypes, including T, B, plasma, and myeloid subsets. In this study, the CIBERSORT algorithm was used to analyse the data of patients with sepsis, to infer the relative proportions of 22 immune infiltrating cells and to perform Spearman correlation analysis on gene expression and immune cell content.

### GSEA

GSEA uses a predefined set of genes, ranks genes according to their degree of differential expression in two types of samples, and then tests whether the predefined gene set is enriched at the top or bottom of the ranking list. In this study, GSEA was used to compare the differences in the KEGG signalling pathway of different groups and to explore the molecular mechanism of core genes in the two groups of patients. The number of substitutions was set to 1000, and the substitution type was set to phenotype.

### Regulatory network analysis of key genes

The transcription initiation process of eukaryotes is very complex and often requires the assistance of a variety of protein factors. Transcription factors and RNA polymerase II form a transcription initiation complex and participate in the process of transcription initiation together. Transcription factors can be divided into two categories according to their functions. The first category is universal transcription factors. When they form a transcription initiation complex with RNA polymerase II, transcription can start at the correct position. A cis-acting element is a sequence flanking a gene that can affect gene expression [10]. Cis-acting elements include promoters, enhancers, regulatory sequences, and inducible elements, which participate in the regulation of gene expression. The cis-acting element itself does not encode any protein but only provides an action site to interact with the trans-acting factor. This analysis was mainly performed by the R package cisTarget, in which we used rcistarget.hg19.motifdb.cisbpont.500 bp for the Gene-motif rankings database.

### Statistical analysis

All statistical analyses were performed in R language (version 3.6). All statistical tests were two-sided, and *p* < 0.05 was considered statistically significant.

## Results

### Differential gene screening

**Fig. 1:**
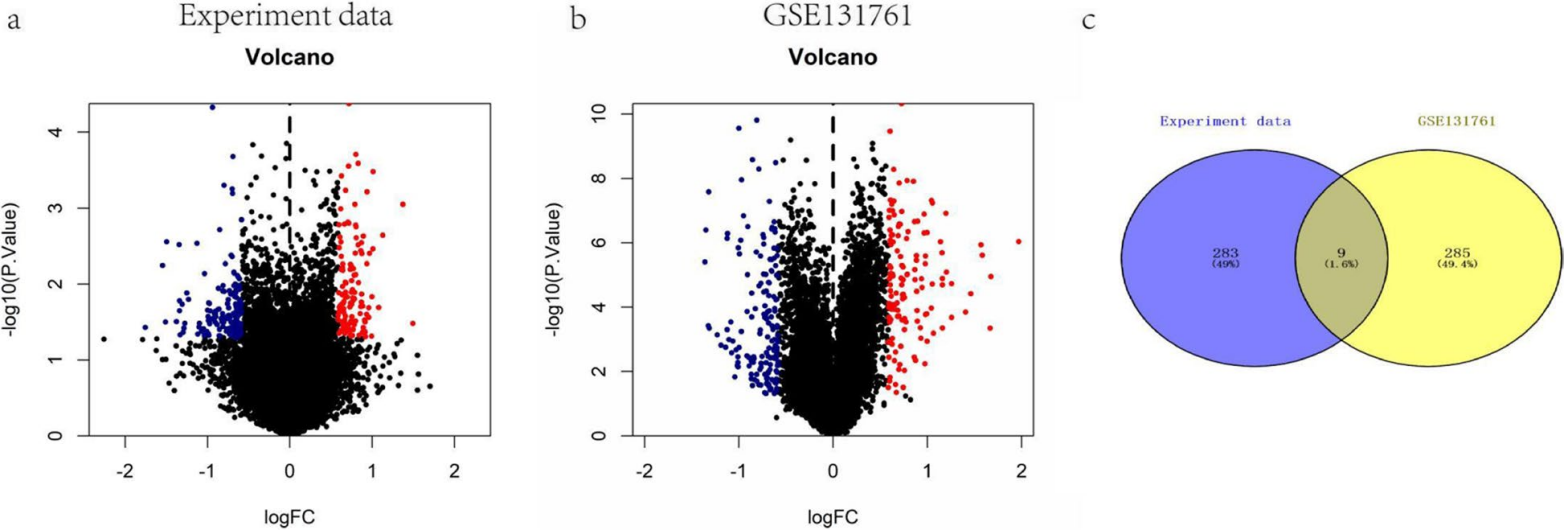
Identification of differentially expressed genes between septic shock patients and controls. **a** and **b** Volcano plot of self-test data and differential expression of GSE131761. Blue indicates differential expression downregulation, red indicates differential expression upregulation, and differential gene screening conditions are *p* < 0.05 & |Log2FC|> 0.585. **c** Venn diagram of differentially expressed genes

A total of 19 patients were included in the self-assessment data, including 10 patients with no septic shock and 9 patients with septic shock. Dataset GSE131761 included expression profile data of 114 groups of patients, including 33 patients with no septic shock and 81 patients with septic shock. We used the limma package to calculate the differentially expressed genes between the two groups of patients. The differential gene screening conditions were *p* < 0.05 & |Log2FC|> 0.585. A total of 292 differentially expressed genes were screened from the self-test data, including 128 upregulated genes and 164 downregulated genes (Fig. 1a). A total of 294 differentially expressed genes were screened in the GSE131761 dataset, including 130 upregulated genes and 164 downregulated genes (Fig. 1b). Then, the differentially expressed genes in the two datasets were intersected, and a total of 9 intersecting genes were obtained (Fig. 1c).

### Functional analysis of GO and KEGG

**Fig. 2:**
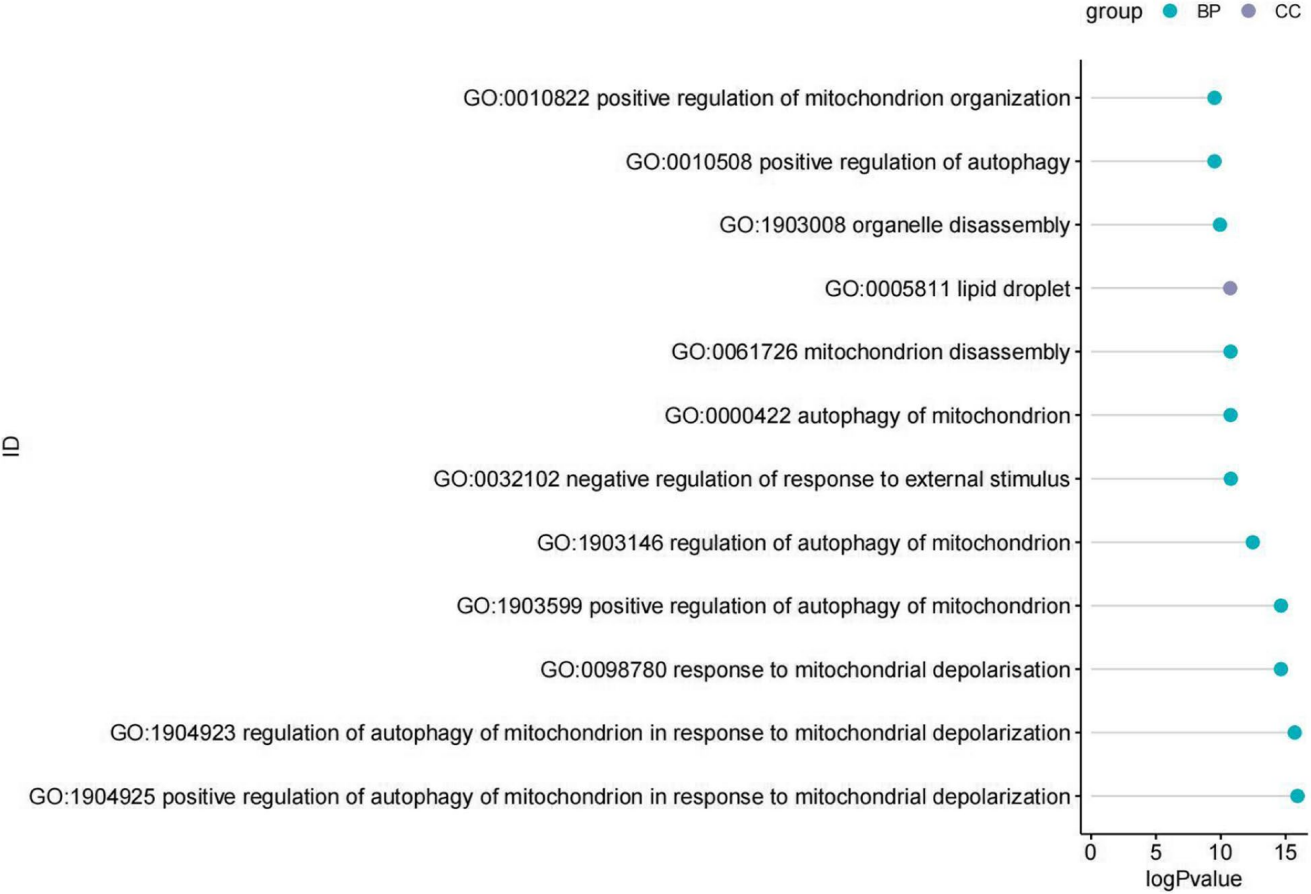
GO enrichment analysis of differentially expressed genes between the septic shock and control groups. GO enrichment results of differentially expressed genes sorted by *P* value

We further performed pathway analysis on these 9 differentially expressed genes [11–13]. The results showed that the differentially expressed genes were mainly enriched in pathways such as the positive regulation of autophagy in mitochondria in response to mitochondrial depolarization, the negative regulation of the response to external stimuli, and the positive regulation of autophagy in mitochondria (Fig. 2).

### Construction of the WGNCA network in septic shock patients

**Fig. 3:**
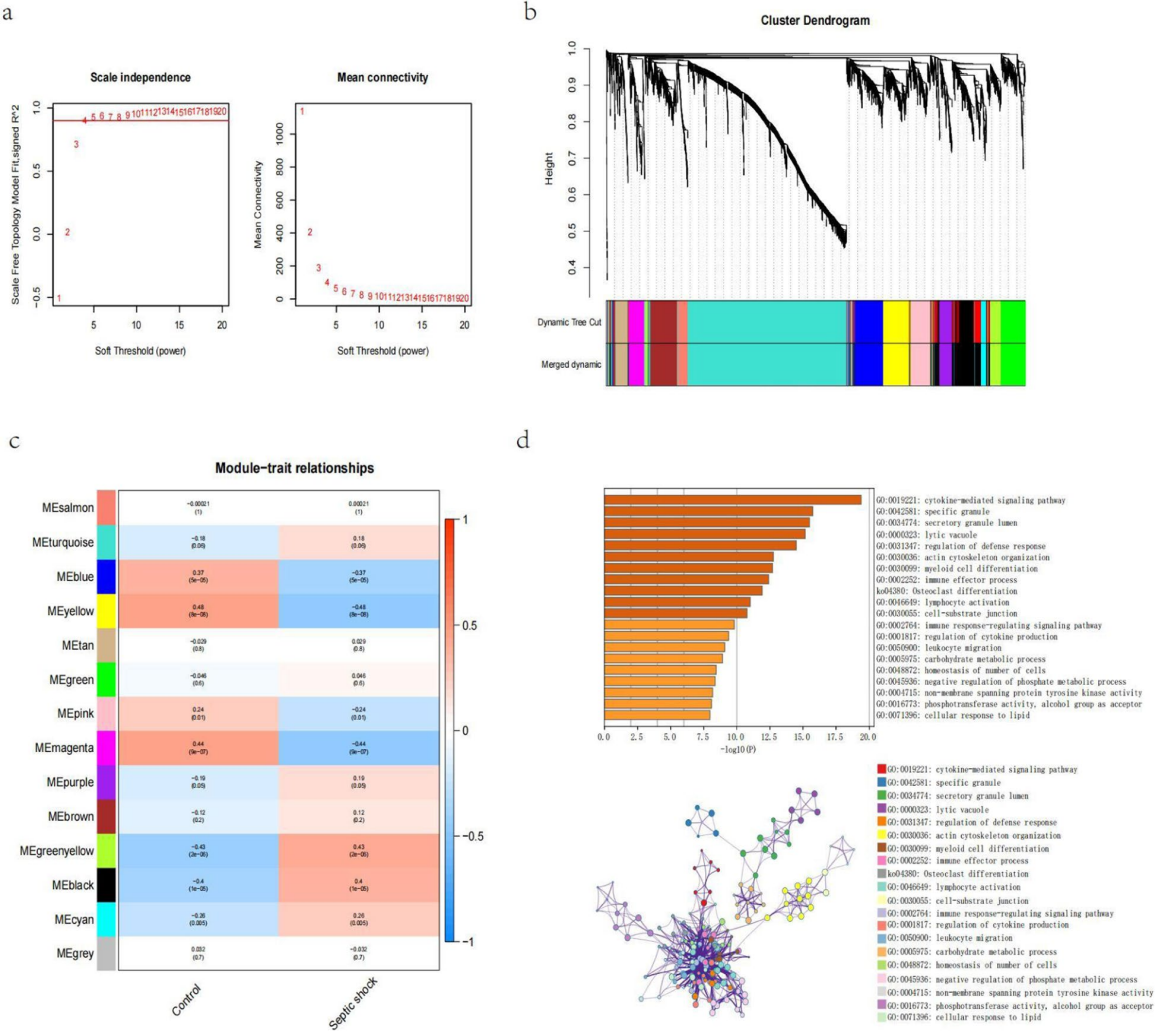
Construction of the WGNCA network in septic shock patients. **a** Scale-free exponent and average connectivity for each soft threshold. **b** Dendrogram of gene clusters, with different colours representing different modules. **c** Heatmap of the correlation between module eigengenes and septic shock. Blue indicates a negative correlation, red indicates a positive correlation, and the yellow module with the highest correlation was selected for subsequent analysis. **d** GO-KEGG enrichment analysis of genes based on the Metascape database

We further constructed a WGCNA network based on the expression profile data of GSE131761 patients to explore the related coexpression network in sepsis. We choose β = 4 (scale-free R2 = 0.9) to build the scale-free network. Then, a hierarchical clustering tree was constructed using dynamic hybrid cutting technology to construct gene modules. Branches represent a series of genes with similar expression data, and each leaf represents a gene on the tree. In addition, 14 modules were built. We found that the yellow module was significantly associated with the disease. We selected the yellow module with the highest correlation with the disease (cor = 0.48, *p* = ((8e − 08)) and performed enrichment analysis through the Metascape database. The results showed that module genes were mainly enriched in cytokine-mediated signalling pathways, specific granules, osteoclast differentiation and other pathways (Fig. 3a-d).

### Screening of septic shock core genes

**Fig. 4:**
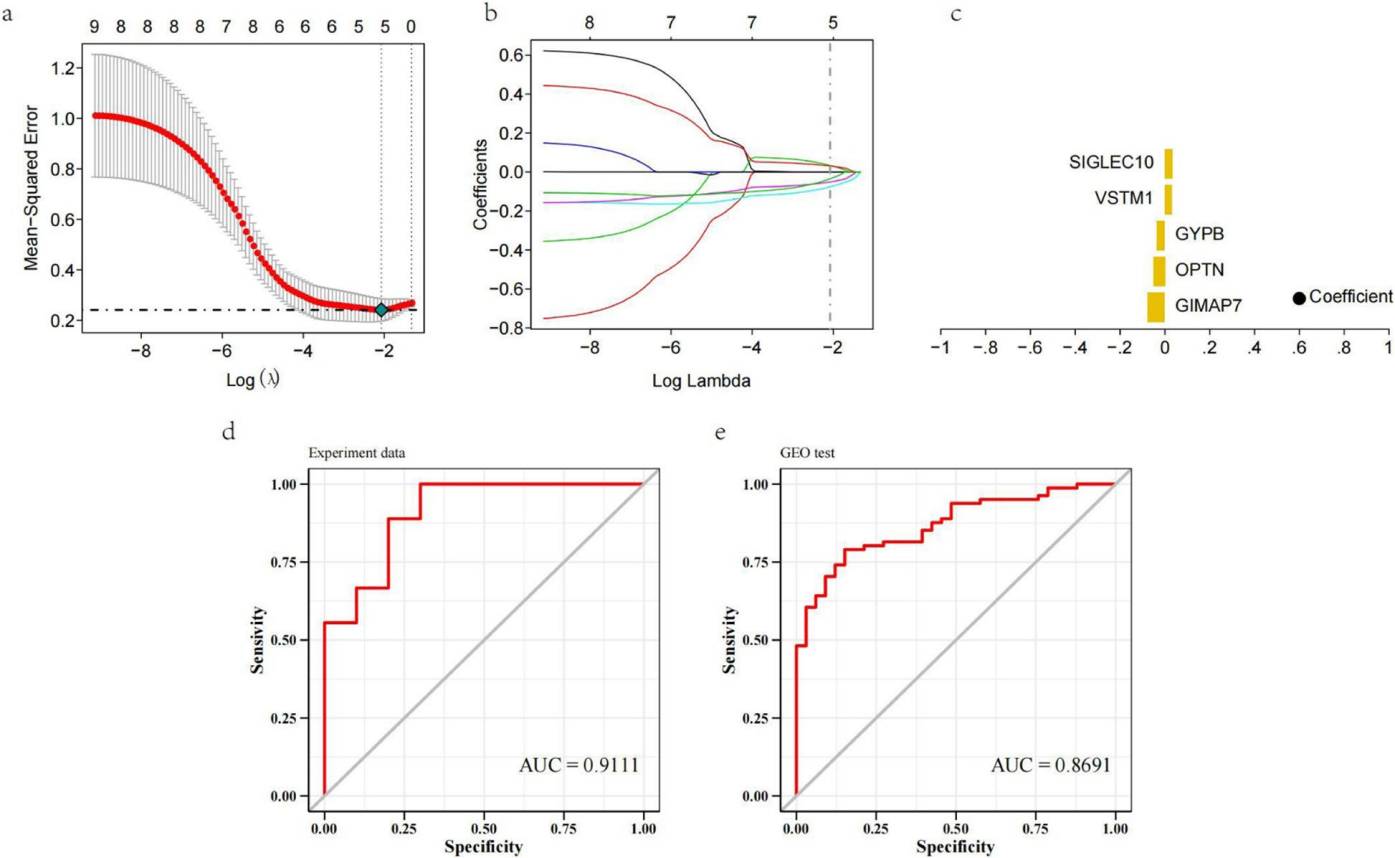
Screening of septic shock core genes. **a** Tenfold cross-validation of tuning parameter selection in the LASSO model. **b** Distribution of LASSO coefficients for differentially expressed genes. **c** Coefficient of the Lasso gene. **d** and **e** The ROC curves of the 5 Lasso genes in the training set and the validation set. The ROC curves are all greater than 0.8, and the model has good predictive performance

To further determine the key genes in the differential gene set, we took the self-test data as the training set and the differential genes in the GSE131761 dataset as the validation set and selected the intersection genes for feature screening through Lasso regression. The results showed that a total of 5 genes were identified by Lasso regression as the characteristic septic shock and as the core genes of the follow-up study; the 5 genes were SIGLEC10, VSTM1, GYPB, OPTN, and GIMAP7 (Fig. 4a-c). In our study, the prediction model was constructed by the lasso algorithm, and the results showed that the prediction model constructed by the 5 genes had good diagnostic performance, and the area under the AUC curve was 0.9111. Using the validation set to further verify the diagnostic model, the results showed that the model had strong diagnostic performance and stability, with an AUC of 0.8691 (Fig. 4d-e).

### Analysis of immune cell infiltration

**Fig. 5:**
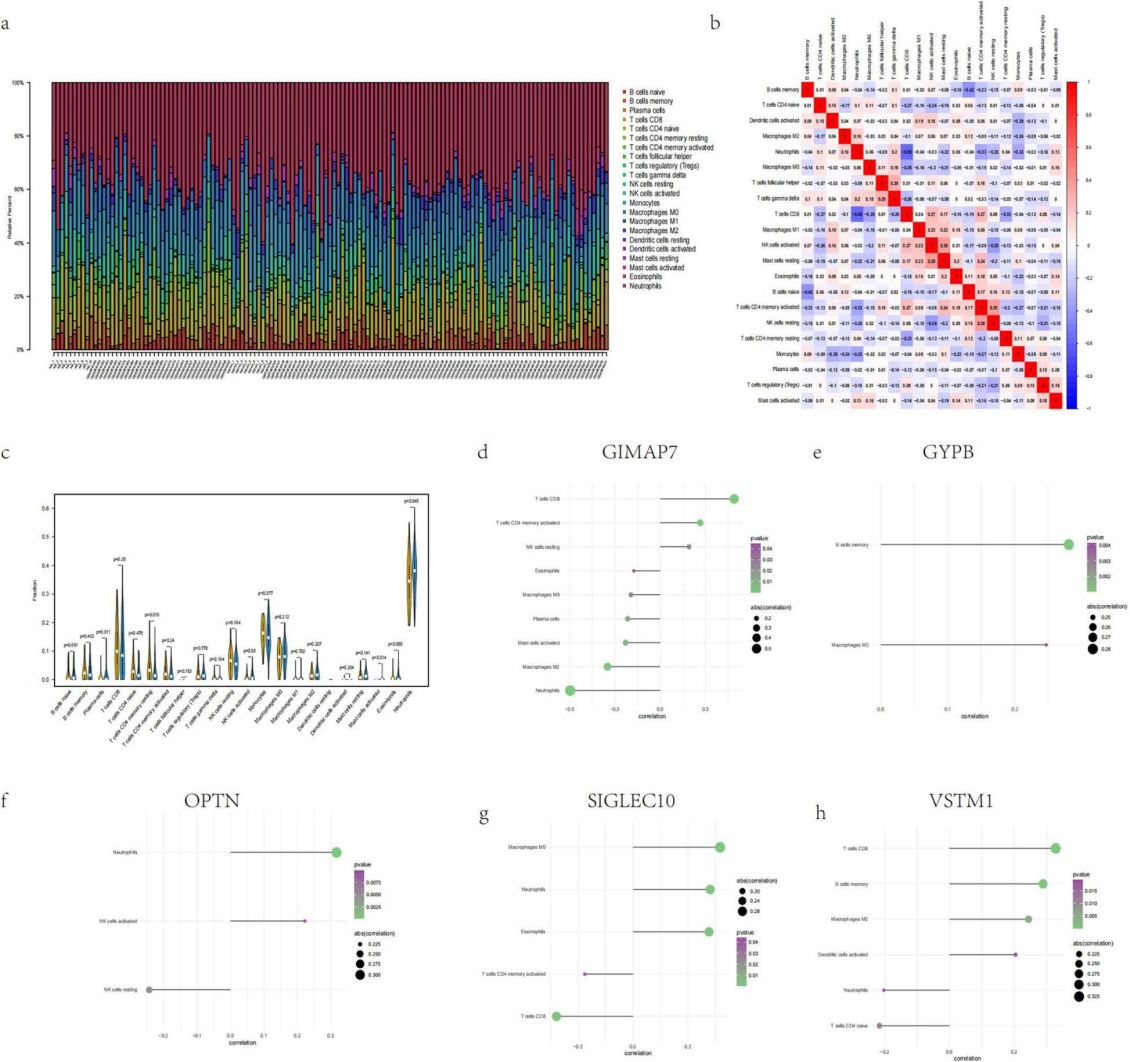
Immune infiltration in all patients with septic shock. **a** Relative percentages of 22 immune cell subsets in all patients. **b** Pearson correlation between 22 immune cells. Blue indicates a positive correlation, and red indicates a negative correlation. **c** Differences in immune cell content between control patients and septic shock patients. Yellow indicates control patients, and blue indicates septic shock patients. *P* < 0.05 was considered statistically significant. **d-h** The Spearman correlation between the expression of five core genes and the content of immune cells. The *P* values of immune cells in the figure are all less than 0.05

The microenvironment is mainly composed of immune cells, extracellular matrix, various growth factors, inflammatory factors and special physical and chemical characteristics, which significantly affect the diagnosis and clinical treatment sensitivity of diseases. By analysing the relationship between core genes and immune infiltration in the “self-test” dataset, we further explored the underlying molecular mechanisms by which core genes affect Sepsis progression (Fig. 5a and b). The results of the study showed that compared with patients with no septic shock, the neutrophils in patients with septic shock were significantly higher than those in normal patients, while resting memory CD4 T cells were lower than those in normal patients (Fig. 5c). Subsequently, we performed Spearman correlation analysis on core genes and immune cells, and five genes had strong correlations with immune cells (Fig. 5d-h).

### Predictive performance of core genes for disease

**Fig. 6:**
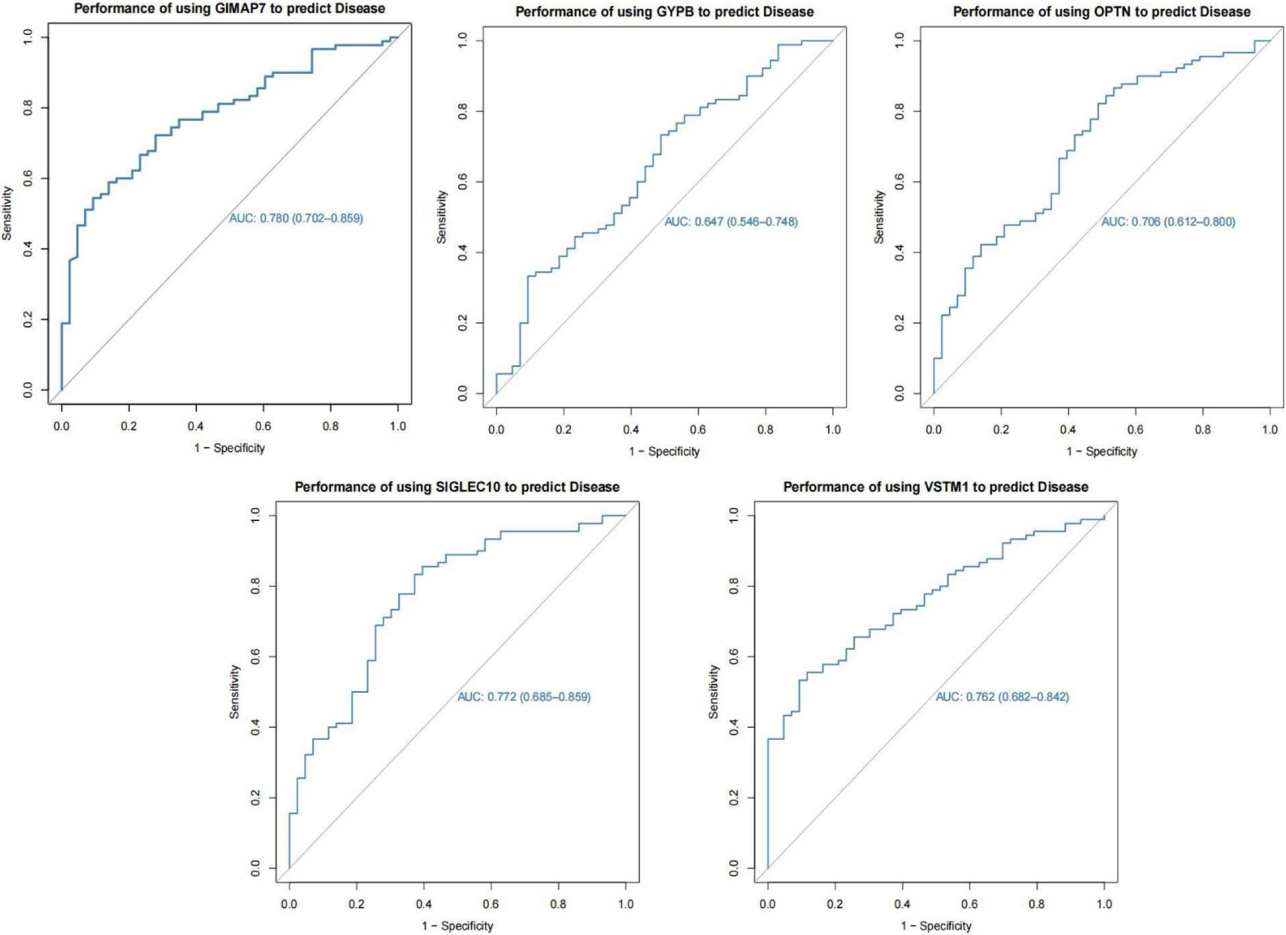
Predictive efficacy of core genes for disease. The ROC curves of control patients and septic shock patients showed that the five core genes had good predictive performance for septic shock

We passed the ROC curve of diagnostic efficacy validation. The higher the AUC value is, the better the predictive performance. The results showed that the AUC values of the five core genes were GIMAP7-AUC: 0.780 (0.702–0.859), GYPB-AUC: 0.647 (0.546–0.748), OPTN-AUC: 0.706 (0.612–0.800), SIGLEC10-AUC: 0.772 (0.685–0.859), and VSTM1-AUC: 0.762 (0.682–0.842). Our analyses suggest that the five core genes can better predict the occurrence and development of the disease (Fig. 6).

### GSEA enrichment analysis

**Fig. 7:**
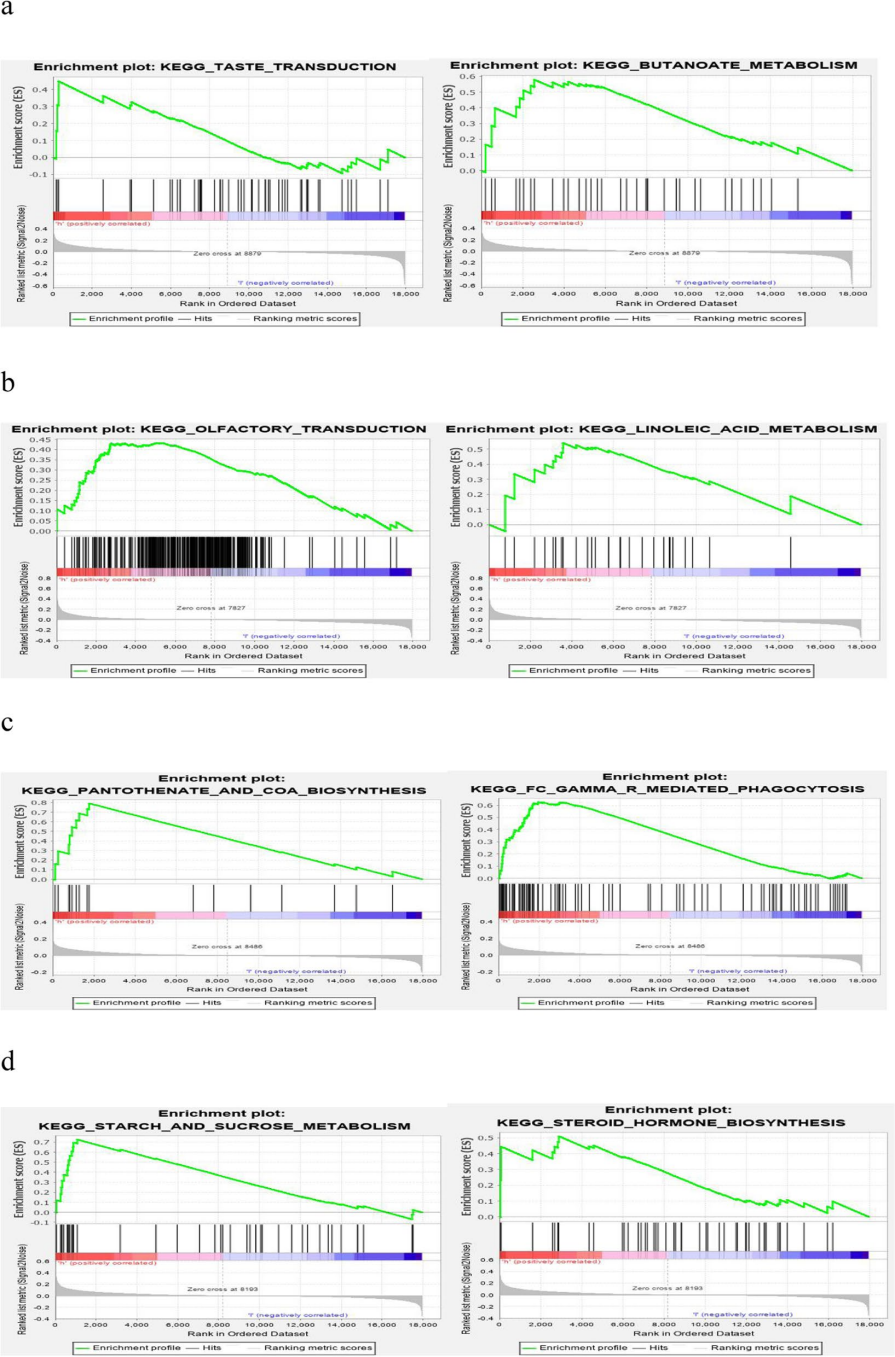
Enrichment map of GSEA. **a** GIMAP7 expression is positively correlated with KEGG_TASTE_TRANSDUCTION, KEGG_BUTANOATE_METABOLISM pathway; **b** GYPB and OPTN expression is positively correlated with KEGG_OLFACTORY_TRANSDUCTION, KEGG_LINOLEIC_ACID_METABOLISM pathway; **c** SIGLEC10 expression is positively correlated with KEGG_PANTOTHENATE_AND_COA_BIOSYNTHESIS, KEGG_FC_GAMMA_R_MEDIATED Positive correlation with KEGG_STARARCH_AND_SUCROSE_METABOLISM, KEGG_STEROID_HORMONE_BIOSYNTHESIS pathway

We next studied the specific signalling pathways enriched by the five core genes and explored the potential molecular mechanisms of the core genes affecting the progression of sepsis. We found significant enrichment in many related pathways through GSEA (Table 1). Some of the highly significant pathways are displayed in a centralized manner (Fig. 7).

**Table 1:** Significant Pathways of Five Genes

Gene	Highly expressed-enriched pathways	
GIMAP7	KEGG_TASTE_TRANSDUCTION	KEGG_BUTANOATE_METABOLISM
GYPB	KEGG_OLFACTORY_TRANSDUCTION	KEGG_LINOLEIC_ACID_METABOLISM
OPTN	KEGG_OLFACTORY_TRANSDUCTION	KEGG_LINOLEIC_ACID_METABOLISM
SIGLEC10	KEGG_PANTOTHENATE_AND_COA_BIOSYNTHESIS	KEGG_FC_GAMMA_R_MEDIATED_PHAGOCYTOSIS
VSTM1	KEGG_STARCH_AND_SUCROSE_METABOLISM	KEGG_STEROID_HORMONE_BIOSYNTHESIS

### Regulatory network analysis of key genes

**Fig. 8:**
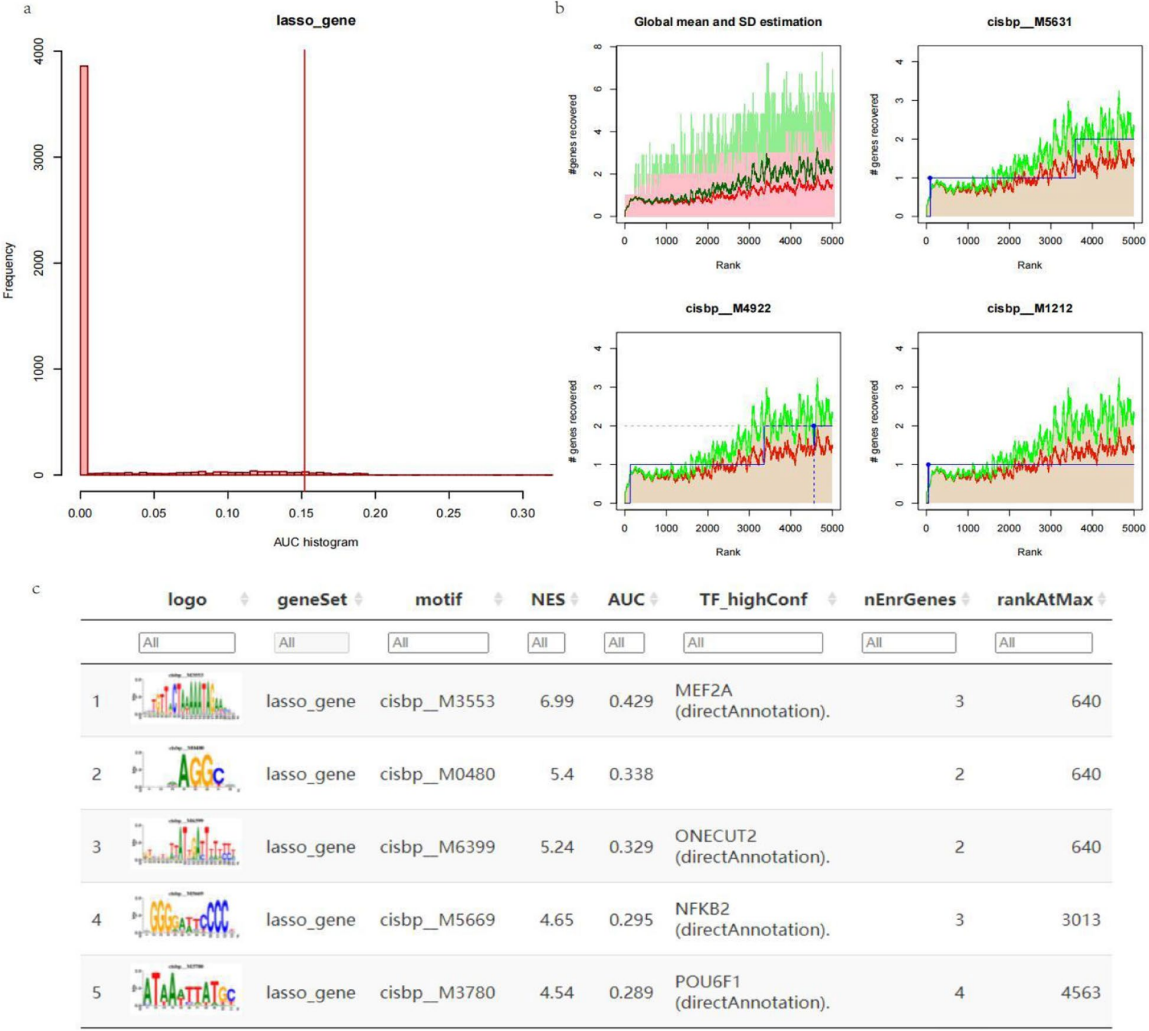
Motif transcriptional regulation analysis of core genes. **a** and **b** The red line is the average of the recovery curves of each motif, the green line is the mean ± standard deviation, and the blue line is the recovery curve of the current motif. The maximum distance point (mean + sd) between the current motif and the green curve is the selected maximum enrichment level. **c** Transcription factors recruited to core genes

We used five core genes for the gene set analysed in this analysis and found that they are regulated by common mechanisms, such as multiple transcription factors, so these transcription factors were enriched using cumulative recovery curves (Fig. 8a and b). The results of the analysis showed that the transcription factor MEF2A was the main regulator in the gene set, which was annotated as cisbp__M3553 by MOTIF. A total of 3 model genes were enriched in this motif. The normalized enrichment score (NES) was 6.99. We display all enriched motifs and corresponding transcription factors for the modelled genes (Fig. 8c).

### Correlation analysis between immune regulatory genes and core genes

**Fig. 9:**
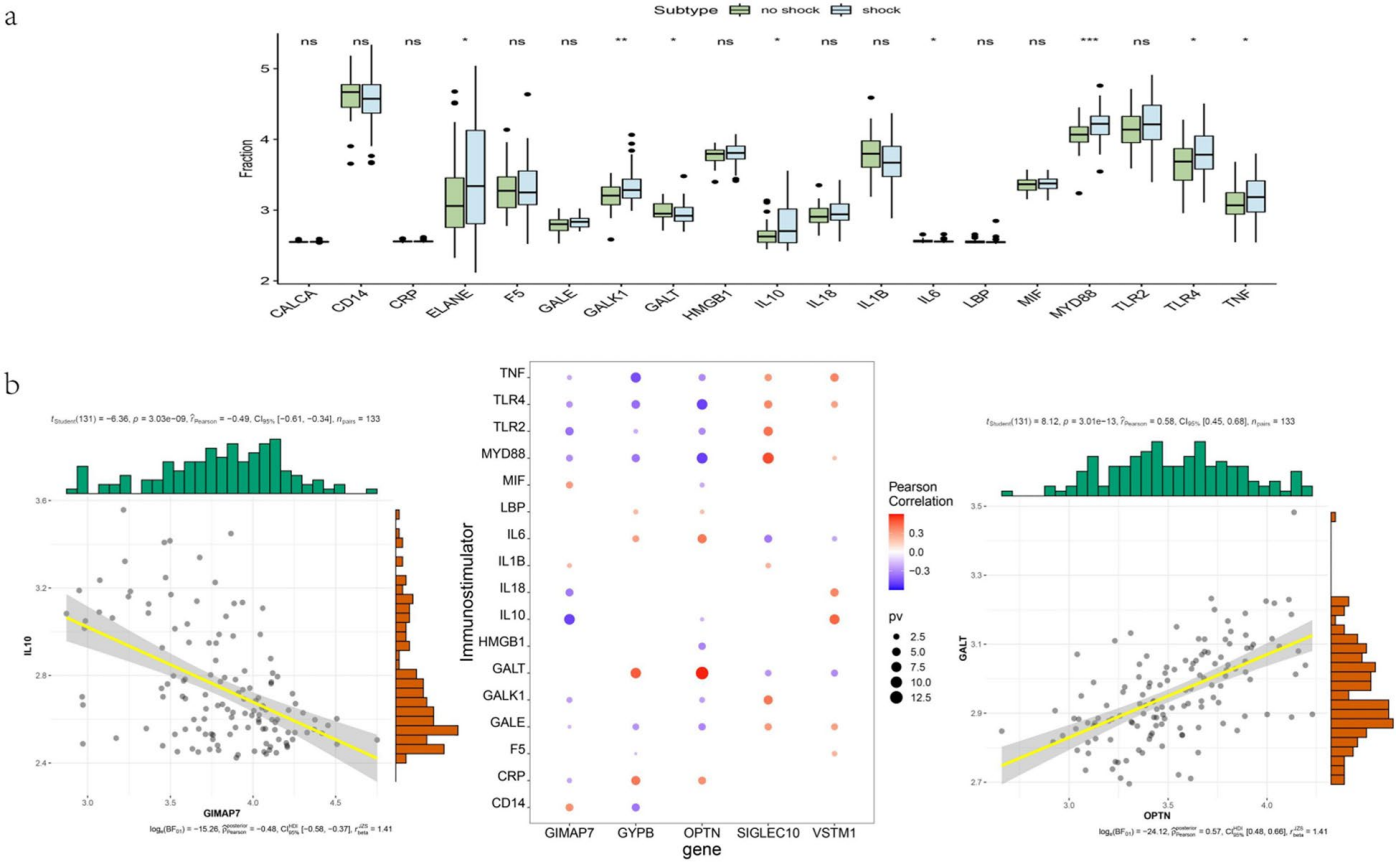
Correlation analysis of septic shock disease regulatory genes. **a** Differences in the expression of septic shock disease-regulating genes; green indicates control patients, and blue indicates diseased patients. **b** Pearson correlation analysis of septic shock disease regulatory genes and core genes. Blue indicates a negative correlation, and red indicates a positive correlation

We performed differential analysis of immune regulatory genes, and the results showed that multiple genes, such as HLA-DMA, HLA-DMB, HLA-DOA, HLA-DPA1, HLA-DPB1, HLA-DPB2, HLA-DQB1, HLA-DQB2, HLA-DRA, etc. There were significant differences between the two groups of patients (Fig. 9a). To explore the relationship between key genes and immune regulation, we conducted correlation analysis on key genes and disease regulation genes. The correlation between key genes and immune regulation genes is shown in the figure. In addition, we searched for sepsis-related regulatory genes through the Genecards database, and the results showed that ELANE, GALK1, GALT, IL10, IL6, MYD88, TLR4, TNF and other genes were significantly different between the two groups of patients, and the key genes were related to sepsis. The correlation of disease-related regulatory genes is shown in Fig. 9b.

## Discussion

Sepsis is an inflammation-induced organ dysfunction, and its pathogenesis includes immune regulation disorders, inflammatory responses, and coagulation disorders. According to statistics, approximately 48.9 million people worldwide suffer from sepsis, of which 11 million die, accounting for 1/5 of the total number of deaths in the world. Although diagnosis and treatment methods, such as mechanical ventilation, fluid therapy and sepsis warning scores have improved continuously, and the morbidity and mortality of sepsis have decreased, they are still the main cause of death in critically ill patients. A cross-sectional survey showed that the incidence of sepsis in intensive care unit (ICU) patients in China was approximately 20%, and the 90-day mortality rate was 35.5%. The fatality rate of the virus remains high. This may be due to the lack of biomarkers for the detection of early sepsis and effective treatment of sepsis [14]. Therefore, understanding the molecular mechanisms of sepsis is necessary for the majority of medical workers to find methods for treating and diagnosing sepsis. In this study, 292 differentially expressed genes were screened out by analysing the self-test data, including 128 upregulated genes and 164 downregulated genes. The GSE131761 dataset screened 294 differentially expressed genes, including 130 upregulated genes and 164 downregulated genes. Then, the differentially expressed genes in the two datasets were intersected, and a total of 9 intersecting genes were obtained. Gene Ontology (GO) and Kyoto Encyclopedia of Genes and Genomes (KEGG) pathway enrichment analyses were performed on these genes to determine the gene function and the associated signalling pathways. The functions of the main signalling pathways focus on mitochondrion in response to mitochondrial depolarization, autophagy of mitochondrion,positive regulation of mitochondrion organization. Zhiyi Jiang found that Ethyl pyruvate protects mitochondria during Sepsis, improves Sepsis outcome by targeting the mitochondrion [15], It indicates that mitochondrial function is related to sepsis.Deng SY and Joseph LC found that we can prevents sepsis by improve mitochondrial function through multiple methods [16, 17].

Finally, 5 main central genes were identified, including SIGLEC10, VSTM1, GYPB, OPTN, and GIMAP7.

Australian scholars have found that most sialic acid-binding immunoglobulin-like lectins (SIGLECs) suppress immune cell function but are expressed at lower levels on human T cells. Soluble CD52 inhibits T-cell signalling by ligating Siglec-10. We examined Siglec-10 expression at the RNA and protein levels in human CD4( +) T cells. These results were consistent with the homeostatic role of Siglec-10 in human CD4( +) T cells [18]. VSTM1 (V-set and 1-containing transmembrane domain) is a novel membrane molecule identified from immunomics, and it has two major isoforms, VSTM1-v1 and VSTM1-v2. VSTM1-v1 is a type I transmembrane protein, and VSTM1-v2 is a typical secreted protein. Compared with VSTM1-v1, it only lacks the transmembrane domain [19]. Some scholars have used whole blood eQTL data from Chinese populations. The identification of SNPs that regulate the expression of the gene encoding SIRL-1, VSTM1, underscores the role of cellular subsets and this inhibitory immune receptor in maintaining skin immune homeostasis [20]. The GYPB gene is mainly studied in immunohaematology research, blood group genomics, etc. [21], OPTN (optineurin) is a macroautophagy/autophagy (hereafter referred to as autophagy) receptor that plays a key role in selective autophagy, which combines autophagy with bone metabolism [22]. GIMAP7 is closely related to the immune process of tumours [23]. Related core genes may affect the immune function of patients with sepsis by upregulating or downregulating their expression, which further affects the development and prognosis of sepsis. For example, the Siglec family is a transmembrane receptor expressed on the surface of immune cells and plays a role in infectious diseases. Regulating the role of immune balance, Siglec-9 regulates the polarization phenomenon of macrophages through the endocytosis of Toll-like receptor 4 (TLR4), which in turn inhibits the action of neutrophils. Siglec-10 inhibits risk-associated molecular patterns (DAMPs), helps T cells to initiate antigen–antibody responses, and reduces the number of B cells to attenuate the inflammatory response [24].

Subsequently, LASSO model expression difference analysis, WGCNA, immune infiltration analysis, GSEA, and key gene regulatory network analysis were performed on these five genes, which were good predictors of disease. Immune infiltration in the sepsis group, according to the ROC curve of diagnostic efficacy validation, indicated that the higher the AUC value, the better the prediction performance. The results showed that the AUC values of the five core genes were GIMAP7-AUC: 0.780 (0.702–0.859), GYPB-AUC: 0.647 (0.546–0.748), OPTN-AUC: 0.706 (0.612–0.800), SIGLEC10-AUC: 0.772 (0.685–0.859), and VSTM1-AUC: 0.762 (0.682–0.842). It is suggested that the five core genes can better predict the occurrence and development of the disease; the above five genes have good diagnostic value in septic shock patients. In conclusion, this study identified DEGs that may be associated with septic shock using bioinformatics research methods. These five genes may serve as potential targets for sepsis diagnosis and treatment, thus providing a scientific basis for the study of the molecular mechanism of sepsis.

## Data Availability

The raw data used to support the fandings of this study are freely available from NCBI datasets. (SUB11250895, https://www.ncbi.nlm.nih.gov/Traces/study/?acc=PRJNA821871)

## Abbreviations

ROC curve: Receiver operating characteristic curve
TLRs: Toll-like receptors
VSTM1: V-set and 1-containing transmembrane domain
OPTN: Optineurin
DAMPs: Damage associated molecular patterns
